# Identification of hub genes and molecular networks involved in alveolar bone resorption based on bioinformatics analysis

**DOI:** 10.1038/s41598-026-48945-x

**Published:** 2026-04-16

**Authors:** Weiwei Lv, Wanyan Zhang, Maolin Zheng, Xiaodan Wang, Dong Lin, Shichen Hu, Xingyu Cai, Ming Zhang, Chenghua Li, Yan Wu

**Affiliations:** 1 Department of Stomatology, Affiliated Hospital of North Sichuan Medical College, Nanchong, 637000 Sichuan China; 2 Department of Stomatology, North Sichuan Medical College, Nanchong, 637000 Sichuan China; 3 Department of Stomatology, Beidaihe Rest and Recuperation Center of PLA, Qinhuangdao, 066100 Hebei China

**Keywords:** Bioinformatics, Osteoclast, Periodontitis, Machine learning, miRNA, Biomarkers, Computational biology and bioinformatics, Diseases, Genetics

## Abstract

Periodontitis is the leading cause of tooth loss, with alveolar bone resorption serving as its fundamental pathological feature. Identifying key regulatory genes and molecular mechanisms is essential for enhancing preventive and therapeutic strategies. Nevertheless, reliable biomarkers and comprehensive regulatory networks have yet to be elucidated. This study aims to systematically identify candidate alveolar bone resorption hub genes (ABRHUB) and their regulatory networks through bioinformatics analysis. Our objective is to establish a bioinformatics foundation for generating hypotheses about the mechanisms underlying periodontitis progression and to identify potential targets for future experimental validation. The GSE16134 dataset extracted from the GEO database was utilized to screen differentially expressed genes in patients with periodontitis and explore the significance of alveolar bone resorption in the prevention and treatment of this condition. Furthermore, these genes were intersected with modular genes screened by weighted gene co-expression network analysis (WGCNA) as well as osteoclast-related genes obtained from molecular characterization databases, and co-expression differential genes (co-DEGs) were obtained. The co-DEGs were subjected to in-depth analysis using various machine learning algorithms. The validation set GSE10334 and qRT-PCR analysis were utilized for double validation, ultimately confirming ABRHUB. The diagnostic value and immune relevance of ABRHUB were further evaluated, and the potential miRNAs and lncRNAs associated with these key genes were predicted using relevant databases. A significant correlation exists between alveolar bone resorption and periodontitis. Through differential analysis of multiple databases and the integration of machine learning techniques, we identified four key ABRHUB genes: NEDD9, P2RX5, CSF1R and NPR3. Subsequently, we validated these genes using the GSE10334 validation set and quantitative reverse transcription polymerase chain reaction (qRT-PCR) analyses, and constructed diagnostic and risk models to elucidate the potential utility of ABRHUB in predicting alveolar bone resorption. Furthermore, the calibration curves we established further validated the accuracy of the model predictions. Ultimately, based on these ABRHUB genes, we constructed a lncRNA-miRNA-mRNA molecular regulatory network, providing a significant bioinformatics foundation for future studies. Four genes associated with osteoclast function, namely NEDD9, P2RX5, CSF1R and NPR3, may serve as potential candidate biomarkers for alveolar bone resorption. Notably, the down-regulation of miR-1260b, miR-1224-5p, miR-3156-5p and miR-4286 may contribute to the progression of periodontitis by promoting the expression of NEDD9, P2RX5 and CSF1R.

**Supplementary Information:**

The online version contains supplementary material available at 10.1038/s41598-026-48945-x.

## Introduction

Periodontitis(PD) represents one of the most prevalent diseases affecting the oral cavity. As of 2021, over 1 billion individuals globally are afflicted by severe periodontitis, a phenomenon that poses a significant challenge to global public health, with this figure steadily increasing over the past few decades^1^. Statistics indicate that the global economic burden of treating periodontitis reached $186 billion in 2019, underscoring its significant impact on global economic security^2^. Alveolar bone loss serves as a critical indicator of periodontitis progression, while uncontrolled alveolar bone resorption constitutes a major factor complicating the treatment of this condition. For individuals suffering from periodontitis, persistent alveolar bone resorption ultimately results in tooth loss, consequently contributing to a growing population of edentulous patients^3^. Tooth loss adversely affects chewing function, aesthetics, psychological well-being and nutritional intake, and may even precipitate systemic diseases, including gastrointestinal, immune, cardiovascular, and neurological disorders^4–6^. Therefore, further elucidation of the molecular regulatory mechanisms of alveolar bone resorption is crucial for developing more effective prevention and treatment strategies.

Osteoclasts represent the principal mediators of alveolar bone destruction in periodontitis. This process is intricately orchestrated by a multifaceted regulatory network comprising inflammatory mediators (e.g., TNF-α, IL-1β), reactive oxygen species (ROS) and emerging mechanisms such as ferroptosis^7–13^. Osteoclast-derived enzymes not only facilitate extracellular matrix degradation, but also intensify periodontal tissue destruction, perpetuating a pathological cycle that exacerbates the clinical progression of periodontitis^14^. Despite the identification of several key regulators through anti-inflammatory, antioxidant and osteoclast-specific strategies, a comprehensive mechanistic understanding remains elusive. Current research is predominantly confined to individual signaling pathways or limited targets, and has yet to comprehensively elucidate the overall architecture of osteoclast-related gene regulatory networks during bone resorption in periodontitis. Crucial hub genes and their interactive landscapes with non-coding RNAs, including miRNAs and lncRNAs, remain insufficiently characterized. This lack of systemic understanding, coupled with the absence of clinically translatable biomarkers (e.g., for early diagnosis or risk stratification), not only hinders a deeper comprehension of the disease mechanisms but also impedes the development of novel targeted therapies and precision diagnostic strategies.

To address these critical bottlenecks and knowledge gaps, the present study employs an integrative bioinformatics framework to systematically identify and validate alveolar bone resorption hub genes (ABRHUB) and their associated molecular interaction networks. Compared with traditional experimental methods, the bioinformatics approach employed in this study enables efficient integration of publicly available gene expression data (e.g., GEO) from periodontitis cases, overcoming sample size limitations to construct a more comprehensive and objective gene expression profile. Specifically, differential gene expression analysis was conducted using the limma package in R. Functional enrichment was performed via clusterProfiler, and co-expression modules were delineated using the Weighted Gene Co-expression Network Analysis (WGCNA) framework. To enhance the precision of hub gene identification, the study integrated machine learning techniques, notably LASSO regression and support vector machine (SVM) algorithms. Furthermore, immune infiltration profiling techniques (e.g., CIBERSORT and ssGSEA) were employed to elucidate the interactive dynamics between ABRHUB and the immune microenvironment of periodontal tissues, with a particular focus on immune cell subsets implicated in osteoclastogenesis. This study employed advanced bioinformatics approaches to comprehensively investigate candidate core molecules and potential regulatory networks that may govern alveolar bone resorption in periodontitis. The findings provide testable hypotheses for elucidating disease pathogenesis and establish a foundation for future experimental studies.

## Results

### Identification of differentially expressed genes in periodontitis

**Fig. 1:**
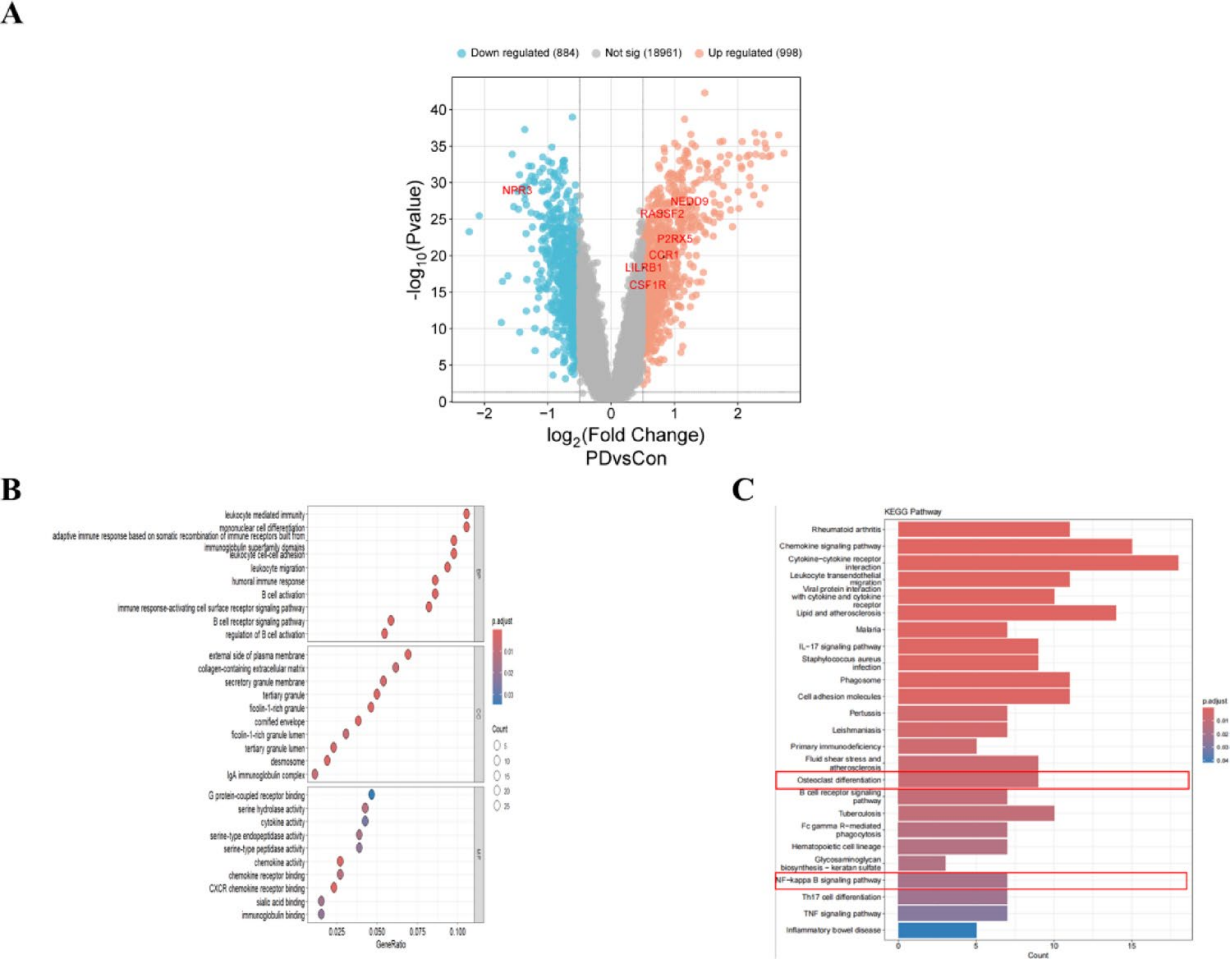
Volcano plot and enrichment analysis of DEGs from the GEO dataset. (**A**) Volcano plot of the GSE16134 dataset; (**B**) GO analysis; (**C**) KEGG analysis^15–17^.

Differential expression analysis of the GSE16134 dataset (241 PD vs. 69 healthy controls) revealed substantial transcriptomic perturbations, identifying a total of 1,882 differentially expressed genes (DEGs) with adjusted P-value < 0.05 and |log_2_FC| > 0.5. Among them, 884 genes were upregulated and 998 genes were downregulated in periodontitis tissues (Fig. 1A). This extensive transcriptomic alteration landscape reflects profound molecular reprogramming of gingival tissues in the context of periodontitis.

GO enrichment analysis revealed that the DEGs were significantly enriched in biological processes and molecular functions including immune cell adhesion, B cell activation, G protein–coupled receptor binding, and CXCR chemokine receptor interactions (Fig. 1B). These results clearly delineate active immune cell recruitment (such as leukocyte infiltration mediated by chemokine signaling), enhanced intercellular interactions (with high expression of adhesion molecules), and significant activation of adaptive immune responses (particularly B cells) at periodontitis lesion sites. This heightened local immune microenvironment is a hallmark of chronic periodontal inflammation and lays the pathological groundwork for subsequent tissue destruction. KEGG pathway enrichment analysis further confirmed that the DEGs were significantly involved in multiple canonical inflammatory and osteoimmune pathways implicated in periodontitis, including the NF-κB, TNF, and IL-17 signaling cascades, as well as the osteoclast differentiation axis (Fig. 1C). Activation of the NF-κB and TNF signaling pathways constitutes the primary driver of pro-inflammatory cytokine storms, including TNF-α, IL-1β, and IL-6. These pathways not only promote tissue inflammation and damage but also critically enhance osteoclastogenesis and bone resorptive function. The involvement of the IL-17 signaling cascade further underscores the pathogenic contribution of Th17 cell–mediated immunity to alveolar bone destruction in periodontitis. The most directly pathologically significant finding was the marked enrichment of the “osteoclast differentiation” pathway. This result unequivocally points to the core cellular mechanism underlying the most destructive pathological outcome of periodontitis—alveolar bone resorption. It indicates that in the periodontal inflammatory environment, the gene networks driving osteoclast formation, differentiation and function are systematically activated.

### WGCNA

**Fig. 2:**
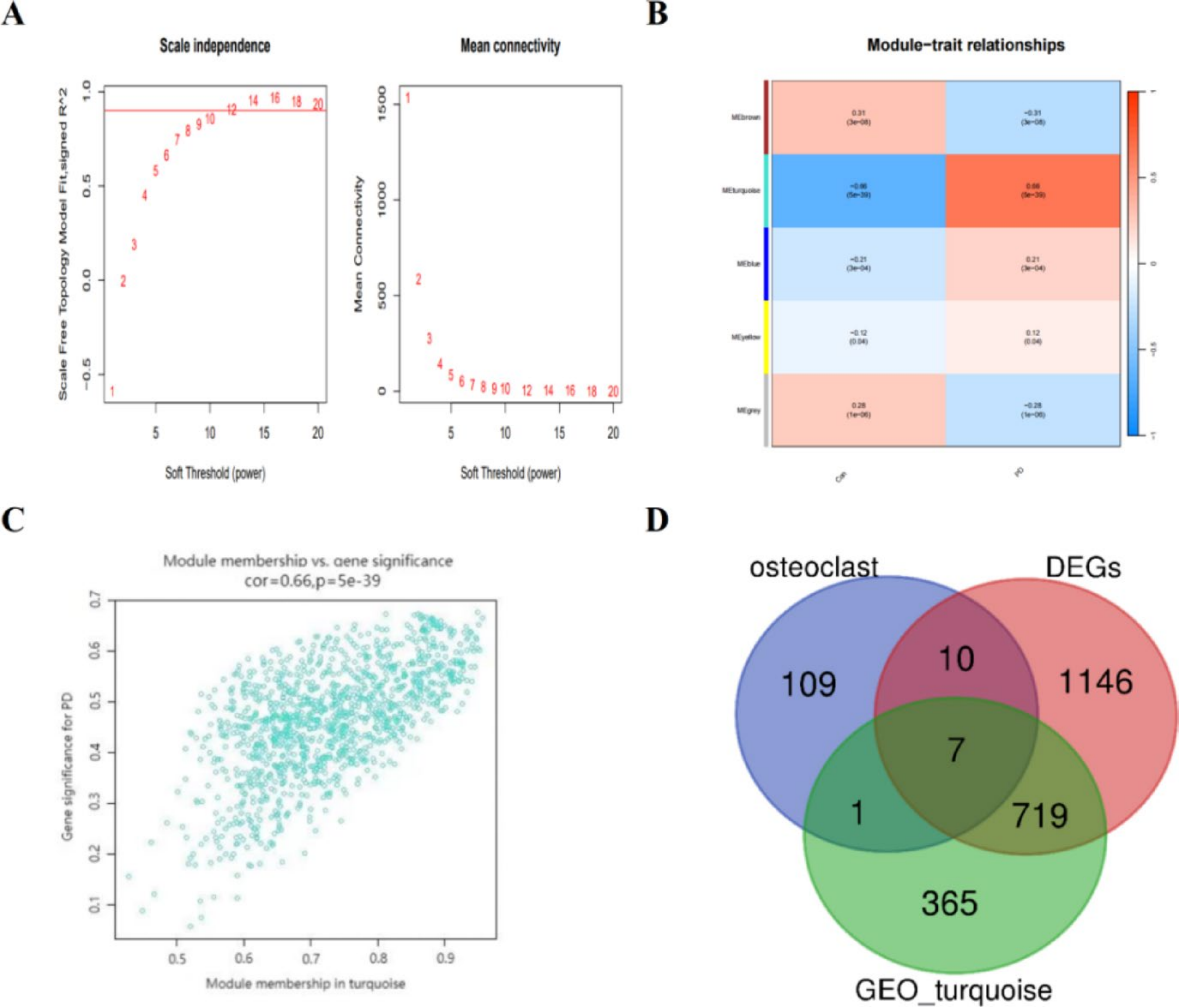
Weighted co-expression network analysis and screening of intersecting genes. (**A**) Determination of the optimal soft threshold; (**B**) heatmap of module-PD association; (**C**) scatterplot of the correlation between module membership (MM) and gene significance (GS) for the genes contained in the turquoise module; (**D**) screening for Co-DEGs.

In the process of conducting WGCNA, five distinct modules were identified with a soft threshold power setting of 12 (Fig. 2A). Among these, the turquoise module comprised 1, 092 genes and exhibited a significant correlation with periodontitis (Fig. 2B and Table S1). In this analysis, the affiliation of the turquoise module demonstrated a strong correlation with the significance of genes associated with periodontitis, yielding a correlation coefficient of 0.66 (Fig. 2C). Consequently, the turquoise module was identified as a critical module for subsequent investigations. Subsequently, 127 osteoclast-related genes were extracted from the molecular characterization database. Through Venn diagram analysis, the intersection of genes within the turquoise module, genes from the molecular characterization database and the DEGs was calculated, resulting in the identification of 7 co-DEGs (Fig. 2D and Table S2).

### Exploring candidate recognition genes using LASSO regression and SVM-RFE algorithm

**Fig. 3:**
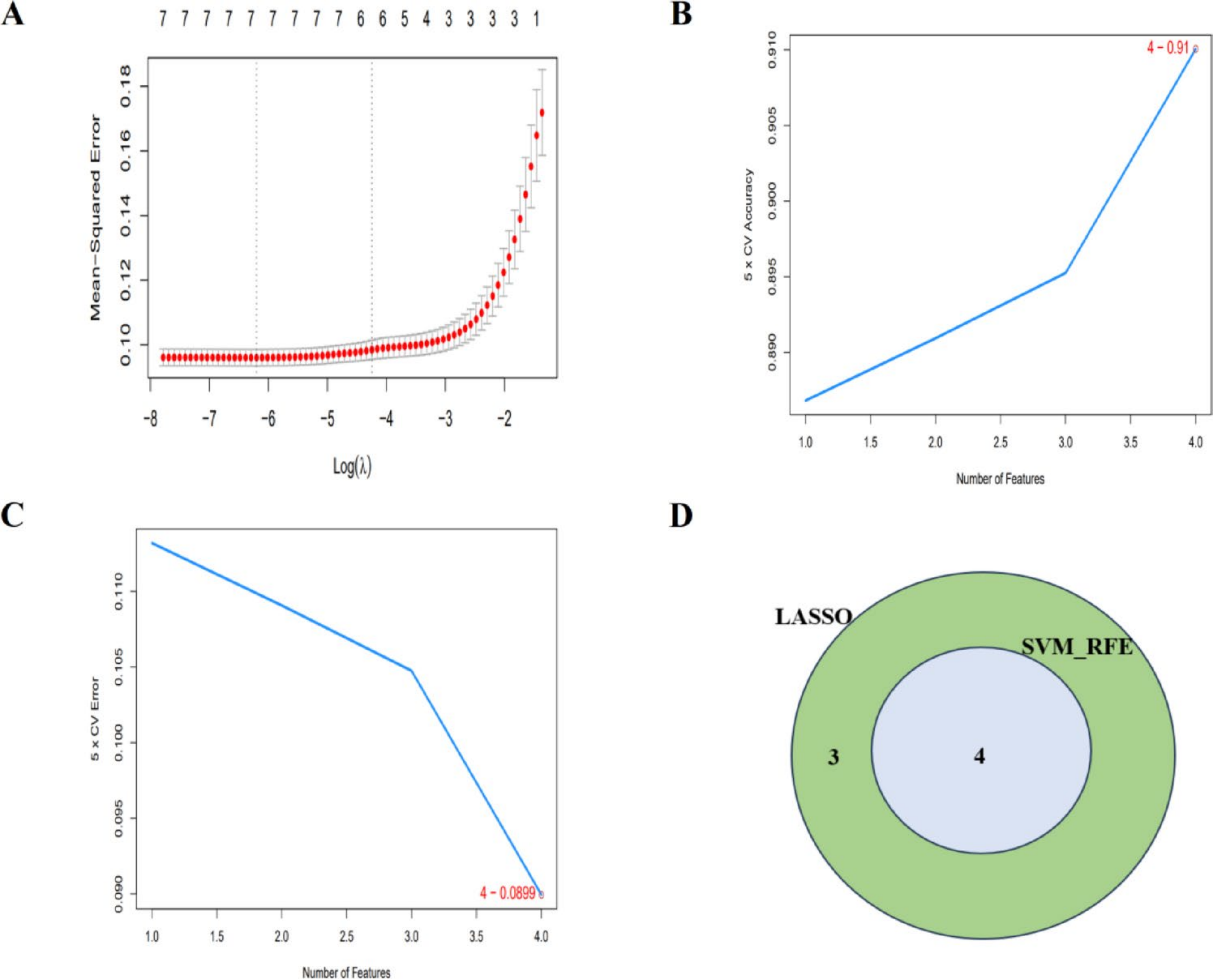
Comprehensive strategy to select the best periodontitis-associated osteoclastic hub genes. (**A**) LASSO logistic regression; (**B**, **C**) SVM-RFE algorithm; (**D**) venn diagram of the intersection of screened diagnostic identifying genes.

Based on these seven genes, machine learning techniques were employed to further identify the co-DEGs. Among these, seven genes were identified through LASSO regression (Fig. 3A), while four genes were identified using the SVM-RFE algorithm (Fig. 3B,C). Ultimately, four osteoclast-related key genes (NEDD9, P2RX5, CSF1R and NPR3) were designated as ABRHUB (Fig. 3D and Table S3).

### Validation of ABRHUB and construction of a PD risk model

**Fig. 4:**
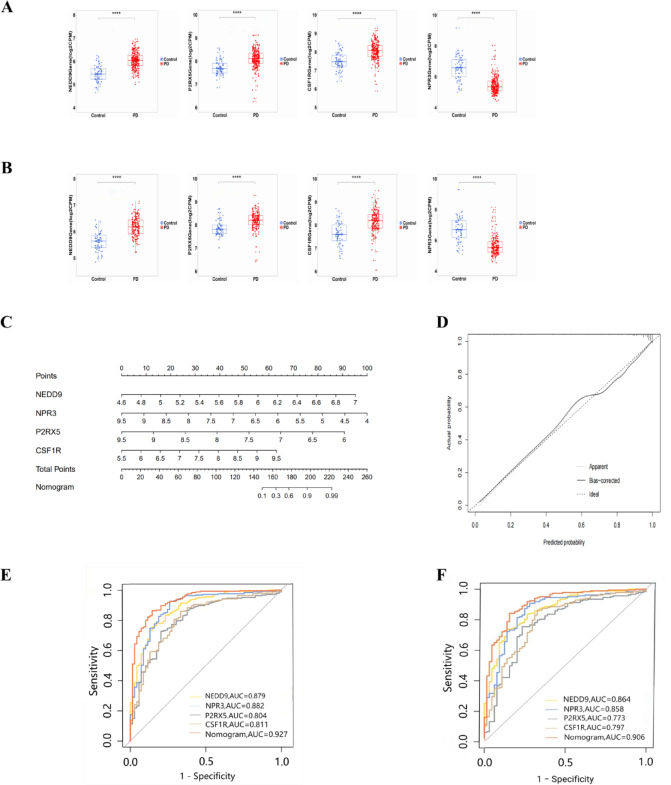
Validation of ABRHUB and construction of a PD risk model. (**A**) ABRHUB expression levels in the training set (GSE16134); (**B**) ABRHUB expression levels in the validation set (GSE10334); (**C**) nomogram model predicting the risk of PD occurrence; (**D**) calibration curves assessing the predictive ability of the nomogram model; (**E**) ROC curve analysis to assess the accuracy of nomogram models in the training set; (**F**) ROC curve analysis to assess the accuracy of nomogram models in the validation set.****, p < 0. 0001.

**Fig. 5:**
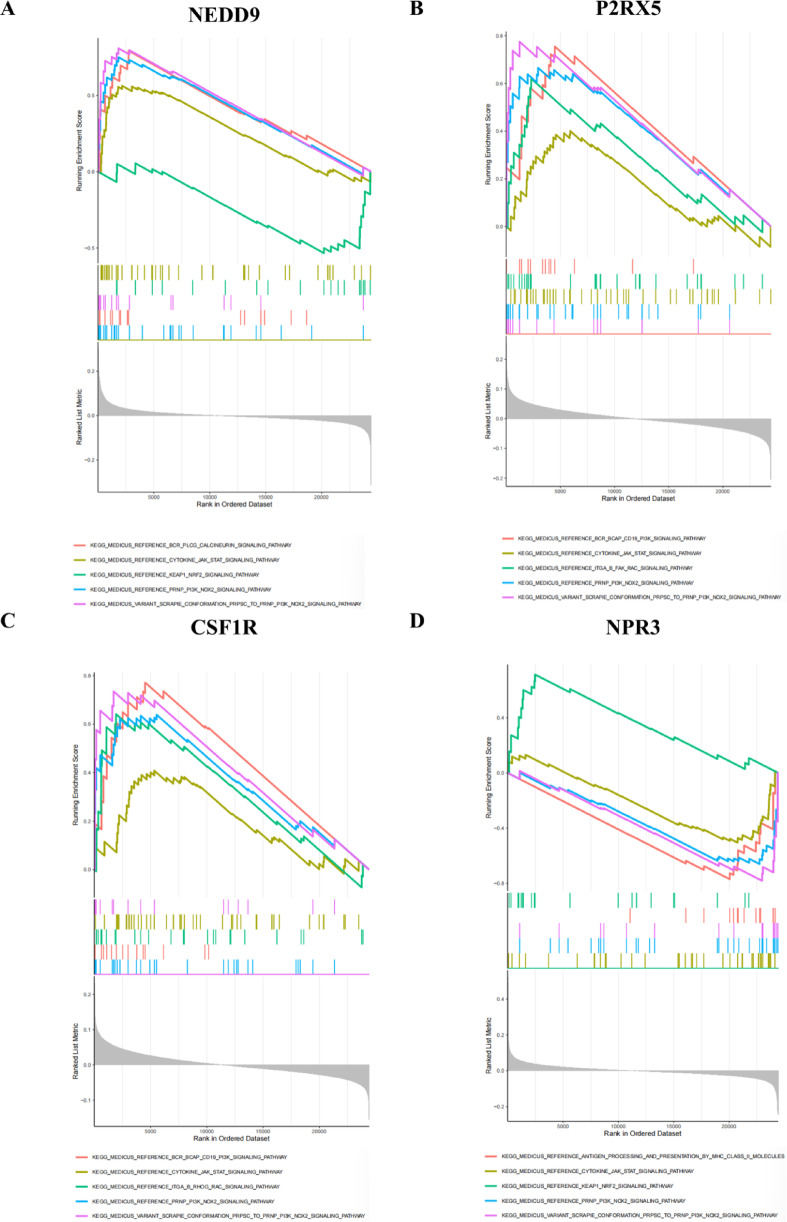
Analysis of major functional enrichment of NEDD9, P2RX5, CSF1R and NPR3 in periodontitis. (**A**) Main enrichment pathway of NEDD9; (**B**) main enrichment pathway of P2RX5; (**C**) main enrichment pathway of CSF1R; (**D**) main enrichment pathway of NPR3.

The expression levels of NEDD9, P2RX5 and CSF1R were significantly up-regulated, while NPR3 was significantly down-regulated in periodontitis samples from the training set GSE16134 compared to the control group (Fig. 4A). This finding was subsequently confirmed through qRT-PCR validation, demonstrating consistency between the bioinformatic predictions and the experimental results (Fig. 8). To further assess the diagnostic potential of these four genes concerning the risk of bone resorption in periodontitis, ROC curve analysis was conducted. The results revealed that the area under the curve (AUC) values for NEDD9, P2RX5, CSF1R and NPR3 in the GSE16134 dataset were 0.879、 0.804、 0.811、and 0.882, respectively, with all ABRHUBs exhibiting AUC values exceeding 80% (Fig. 4E). The validation dataset GSE10334 was employed to confirm the results, and the expression levels of ABRHUB were consistent with those observed in the training set (Fig. 4B). Further ROC analysis of the validation set indicated that the AUC values for NEDD9, P2RX5, CSF1R and NPR3 were 0.864, 0.773, 0.797 and 0.858, respectively (Fig. 5F). Notably, the AUC values for all ABRHUBs exceeded 75%, underscoring the high accuracy of NEDD9, P2RX5, CSF1R and NPR3 as identified genes.

Concurrently, our study developed a nomogram model based on ABRHUB to illustrate their effects on periodontitis (Fig. 4C). The results from the calibration curves indicated that the periodontitis-based prediction model exhibited robust predictive ability, further validating the utility of the model in disease assessment (Fig. 4D). Additionally, the AUC value derived from ROC analysis reached 0. 938, indicating that the model possessed a significantly high accuracy in predicting periodontitis (Fig. 4E). This series of findings suggests that these four ABRHUBs may play a pivotal role in the pathogenesis of periodontitis, offering a significant theoretical foundation for a deeper understanding of the mechanisms underlying the development of periodontitis.

### Gene set enrichment analysis of ABRHUB

Utilizing GSEA, we further investigated the pathways associated with the four ABRHUBs. The results indicated that ABRHUB demonstrated significant enrichment in the JAK/STAT signaling pathway and the PI3K/NOX2 pathway, suggesting that these genes may play crucial roles in cellular signaling. Furthermore, the analysis revealed a negative correlation between NEDD9 and the KEAP1/NRF2 pathway, whereas NPR3 exhibited a positive correlation with this pathway. These findings further elucidate the distinct roles played by the various ABRHUBs within this pathway. Overall, the GSEA results indicate a robust association between ABRHUB and both infection and oxidative stress, suggesting that these genes may be biologically significant in regulating physiological responses and managing environmental stressors. Consequently, ABRHUB may play important roles in several critical signaling pathways, particularly biological processes related to infection and oxidative stress, warranting further exploration in subsequent studies (Fig5A–D)

### Immune cell infiltration analysis

**Fig. 6:**
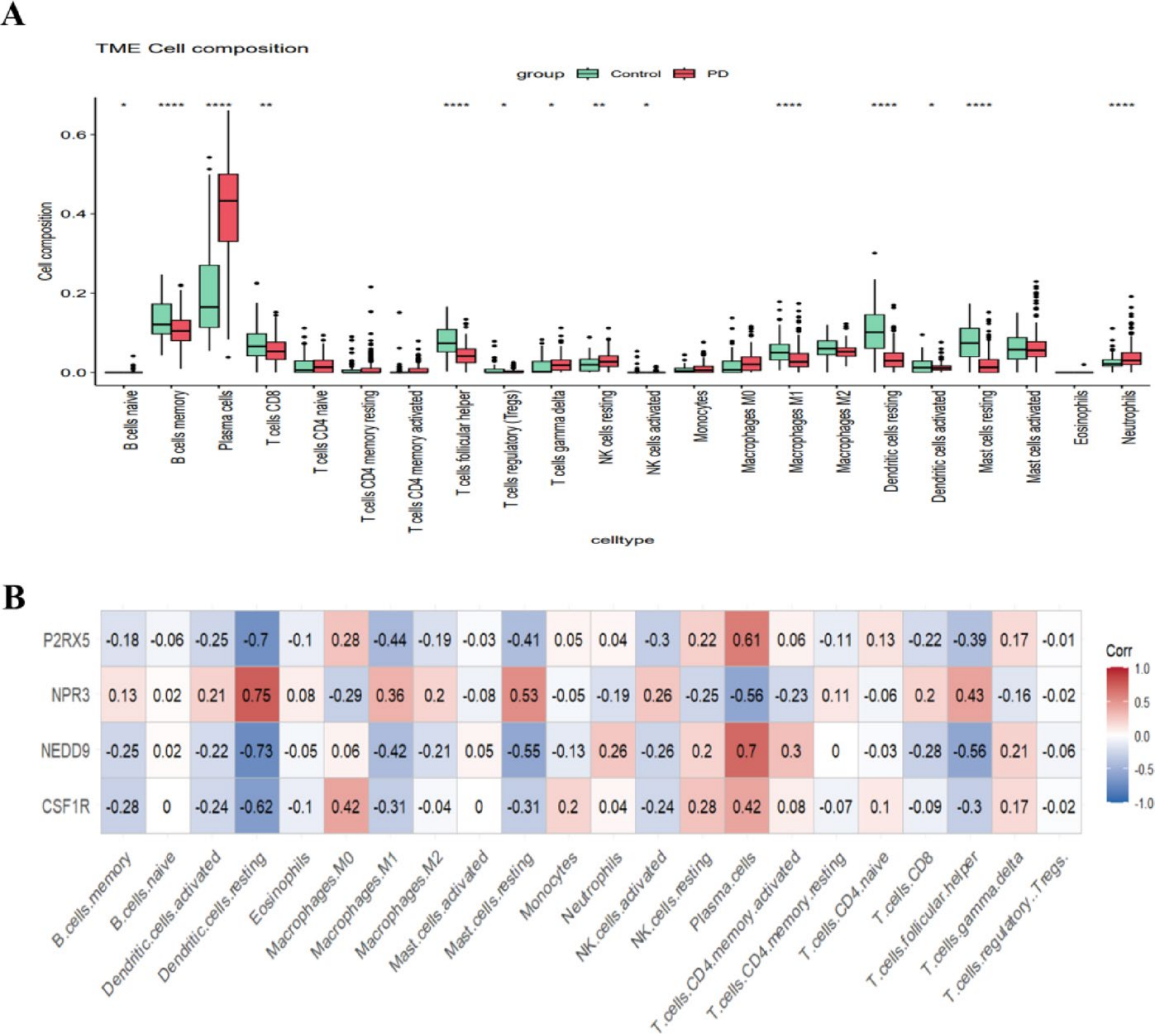
Characterization of immune infiltration in periodontitis and control groups. (**A**) Differential analysis of immune cells in control (Green) and periodontitis (Red) groups; (**B**) heatmap of the correlation between NEDD9, NPR3, P2RX5 and CSF1R and immune cell infiltration.

Given that the immune-inflammatory response is recognized as a critical regulator of alveolar bone resorption in periodontitis, we employed the CIBERSORT algorithm to conduct a comprehensive analysis of the correlation between 22 immune cell types and ABRHUB. Utilizing box plots and heatmaps, we identified a significant correlation between ABRHUB expression levels and plasma cells (Fig. 6A,B). These findings indicate that aberrant expression of genes such as NEDD9, NPR3, P2RX5 and CSF1R significantly influences immune activity associated with periodontitis. Consequently, these genes may be regarded as important markers for elucidating the association between ABRHUB and the immune response, warranting further investigation.

### Predicting targeted miRNAs/lncRNAs

**Fig. 7:**
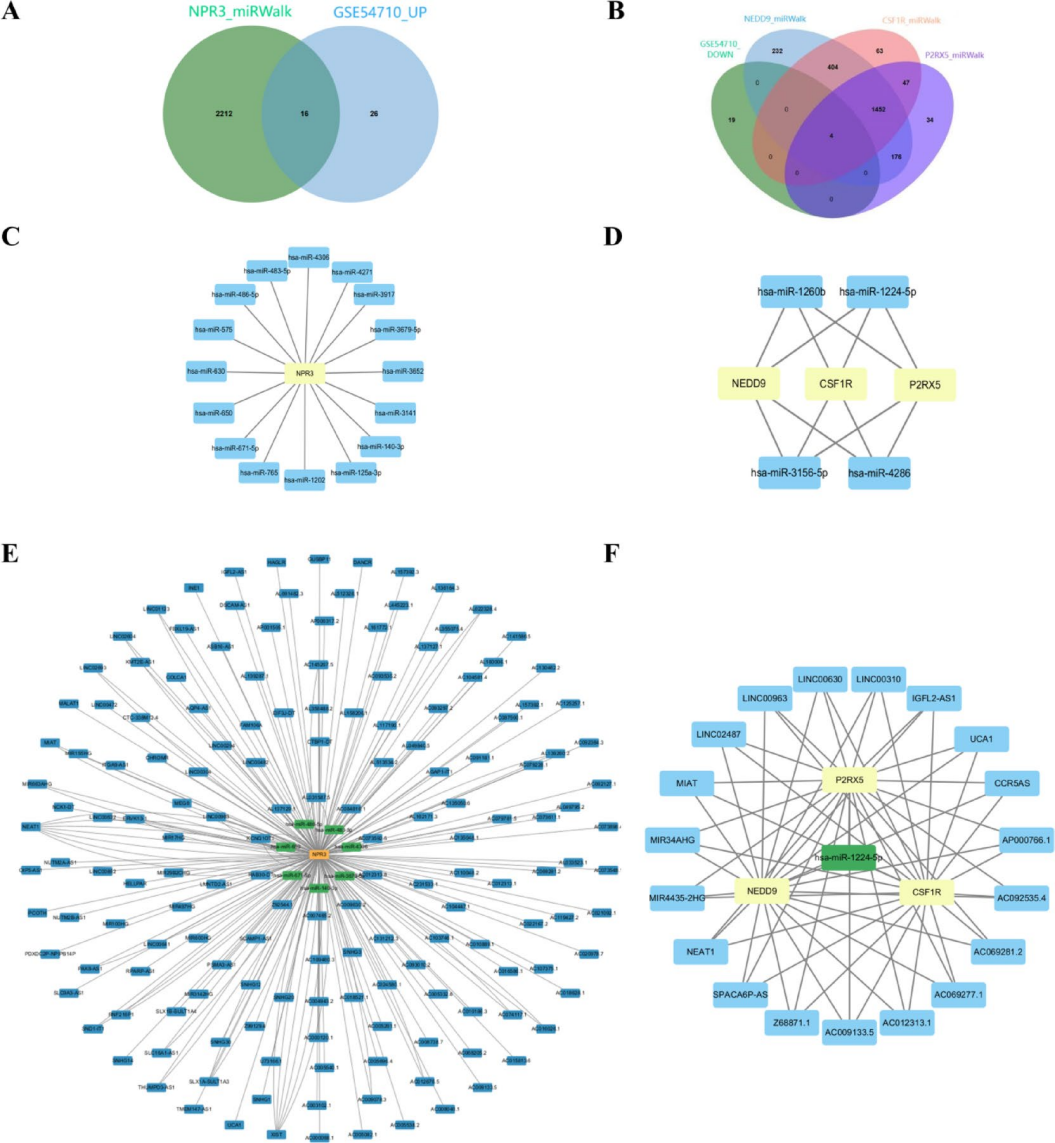
lncRNA-miRNA-mRNA molecular regulatory networks. (**A**) Screening of venn diagrams for the intersection of NPR3-Predicted miRNAs and up-regulated miRNAs expressed in the GSE54710 dataset; (**B**) screening of venn diagrams for the intersection of NEDD9, CSF1R and P2RX5-predicted miRNAs and down-regulated miRNAs expressed in the GSE54710 dataset; (**C**, **D**) potential miRNAs targeted by ABRHUB; (**E**, **F**) construction of ABRHUB’s lncRNA-miRNA-mRNA molecular regulatory network.

To thoroughly investigate the potential upstream targets of ABRHUB, we utilized the miRWalk database to predict miRNAs targeting ABRHUB, subsequently validating these predictions with the periodontitis-related miRNA dataset GSE54710. The results indicated that the miRWalk database predicted 2, 228 miRNAs targeting NPR3, which were then intersected and compared with highly expressed miRNAs in GSE54710, ultimately identifying 16 key miRNAs associated with osteoclasts (Fig. 7A,C and Table S4). Additionally, miRWalk predicted that the number of miRNAs targeting NEDD9, CSF1R and P2RX5 were 2, 268, 1, 970 and 1, 713, respectively. By comparing these with low-expressed miRNAs in GSE54710, we ultimately identified four co-expressed miRNAs, including miR-1260b, miR-1224-5p, miR-3156-5p and miR-4286 (Fig. 7B,D and Table S5). To construct the lncRNA-miRNA-mRNA molecular regulatory network, we screened for relevant lncRNAs associated with ABRHUB using the ENCORI database. Among these, 7 miRNAs targeting NPR3 (including miR-436-5p, miR-483-5p, miR-4306, miR-3679-5p, miR-140-3p, miR-671-5p, and miR-650) were identified, predicting a total of 201 lncRNAs (Fig. 7E). Similarly, for the co-expressed miRNA targeting NEDD9, CSF1R and P2RX5 (i.e., miR-1224-5p), we predicted 19 additional lncRNAs (Fig. 7F). Our comprehensive analysis of the interrelationships between miRNAs and lncRNAs provides valuable potential targets to elucidate the molecular mechanisms underlying periodontitis, which holds significant research implications.

### Validation of ABRHUB in clinical samples

**Fig. 8:**
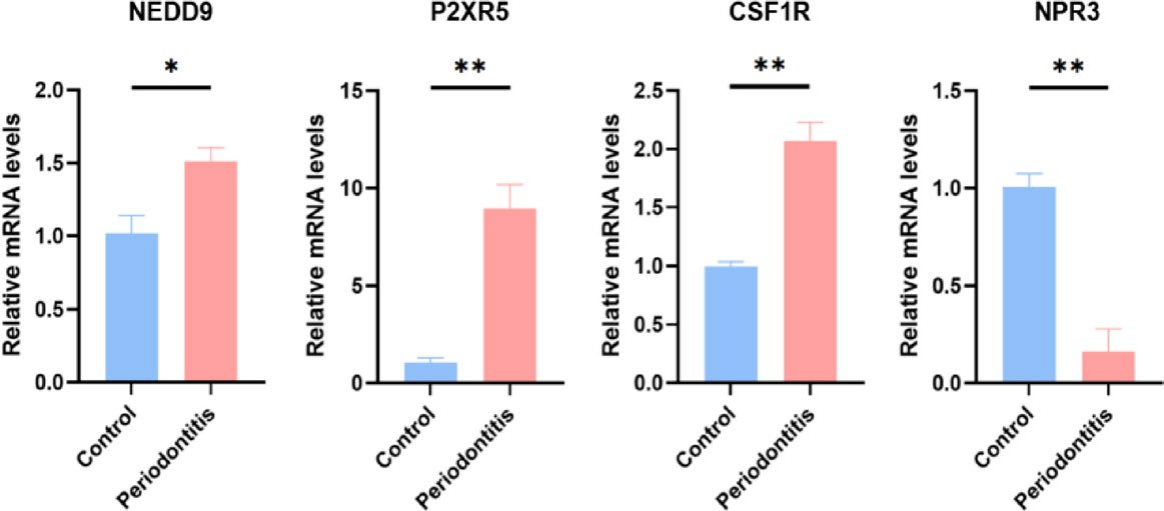
mRNA expression levels of ABRHUB in periodontal patients and healthy controls. *, p < 0. 05;**, p < 0. 01.

We experimentally validated ABRHUB through qPCR analysis conducted on healthy controls and individuals with periodontitis. The results revealed that NEDD9, NPR3 and P2RX5 were significantly up-regulated in periodontitis samples. In contrast, NPR3 was significantly down-regulated (Fig. 8). These validation results were consistent with the findings from the transcriptomic analysis data.

## Discussion

Periodontitis, a globally prevalent chronic inflammatory disorder, is typified by progressive and uncontrolled alveolar bone resorption, which not only culminates in tooth loss and impaired quality of life but also constitutes a significant socioeconomic burden^18,19^. Although osteoclasts play a central role in bone resorption, mechanisms such as inflammation and oxidative stress are involved. However, there is a significant gap in systematically analyzing the osteoclast-related gene regulatory network and its key hub genes that drive bone resorption in periodontitis. Through the integrative application of advanced bioinformatic methodologies to publicly available transcriptomic datasets, we successfully delineated four osteoclast-associated hub genes (termed ABRHUB): NEDD9, NPR3, P2RX5 and CSF1R. These discoveries offer fresh mechanistic insights into periodontitis-associated bone resorption and establish a molecular framework for the rational development of innovative diagnostic strategies and precision therapeutics.

The principal contribution of this study resides in the systematic elucidation of NEDD9, NPR3, P2RX5 and CSF1R as central regulatory determinants of alveolar bone resorption in periodontitis. These genes demonstrated robust diagnostic efficacy (AUC > 75%) across independent transcriptomic cohorts (GSE16134 and GSE10334), underscoring their potential utility as clinically relevant biomarkers. The observed upregulation of NEDD9 aligns with prior reports implicating its involvement in osteoarthritis and osteoclastogenesis, where it promotes bone resorption through the TGF-β/SMAD2/3 signaling cascade^20–23^. NPR3 downregulation unveils a putative regulatory axis, whereby the removal of C-type natriuretic peptide (CNP) indirectly potentiates osteoclast activity, thereby opening new avenues for elucidating CNP’s role in periodontal bone homeostasis^24,25^. The upregulation of P2RX5 is closely associated with ATP sensing, production of pro-inflammatory factors (such as IL-1β), and osteoclast maturation, highlighting the importance of purinergic signaling in periodontal bone destruction and providing a theoretical basis for targeting the ATP-P2 × 5 axis^26–28^. Elevated CSF1R expression, along with its modulation of early osteoclast differentiation via the NF-κB axis, reinforces its central role in inflammation-driven bone resorption and highlights CSF1R blockade as a promising therapeutic avenue^29,30^. Collectively, these findings enrich our mechanistic understanding of the intricate gene regulatory circuits underpinning periodontal bone resorption, bridging canonical signaling cascades (e.g., TGF-β, NF-κB) with pivotal effector genes, and illuminating previously unrecognized regulatory nodes (e.g., NPR3-CNP, P2 × 5-ATP).

GSEA further implicated the ABRHUB genes in several key signaling pathways. NEDD9, P2RX5 and CSF1R exhibit positive correlations with the JAK/STAT signaling axis. Prior studies have shown that their inhibition mitigates periodontitis-associated bone resorption, reinforcing their candidacy as therapeutic targets^31^. NEDD9 and NPR3 displayed inverse associations with the NRF2/HO-1 antioxidative stress pathway—negative and positive, respectively—underscoring the critical role of redox regulation in osteoclastic bone resorption. Immune cell infiltration profiling demonstrated a pronounced enrichment of plasma cells in periodontitis lesions, exhibiting positive correlations with NEDD9, P2RX5 and CSF1R, and a negative correlation with NPR3. These findings align with the role of plasma cells in promoting bone resorption through the secretion of pro-osteoclastogenic factors^32,33^. The results suggest that ABRHUB genes may orchestrate both the direct regulation of osteoclast activity and the indirect modulation of the immune microenvironment, particularly through plasma cell infiltration, thereby exacerbating alveolar bone destruction.

We identified a repertoire of candidate regulatory miRNAs (e.g., miR-1260b, miR-1224-5p) and lncRNAs (e.g., NEAT1) with putative interactions involving ABRHUB genes. These in silico-predicted non-coding RNA networks, such as miR-1224-5p targeting NEDD9/P2RX5, offer mechanistic insights into the post-transcriptional regulation of ABRHUB gene expression. Although certain miRNAs (e.g., miR-1260b) have demonstrated bone-resorption-suppressive properties in foundational studies, the ABRHUB–miRNA–lncRNA regulatory circuits proposed herein warrant rigorous experimental validation and in-depth mechanistic dissection^34,35^.

The ABRHUB identified in this study demonstrate potential as candidate biomarkers, subject to rigorous future validation. NEDD9, NPR3, P2RX5, CSF1R, and their combinations exhibit promising diagnostic capability in silico, evidenced by high AUC values. However, before any clinical application can be considered, future research must prioritize: (1) validating their efficacy as diagnostic/prognostic biomarkers in larger-scale, multi-center prospective cohorts; (2) developing and testing convenient detection methods (e.g., saliva/gingival crevicular fluid-based assays). (3)longitudinal study designs following disease progression or treatment response would provide valuable insights into the temporal dynamics of ABRHUB expression and their utility as prognostic biomarkers. Furthermore, these genes represent attractive candidates for therapeutic targeting, but this potential is entirely contingent on successful functional validation studies. Future directions include: (1) investigating NEDD9-mediated TGF-β/SMAD2/3 signaling in osteoclastogenesis using gene knockout models; (2) exploring strategies to modulate CNP levels or NPR3 activity; (3) developing P2 × 5 receptor antagonists and testing in preclinical periodontitis models; (4) evaluating existing CSF1R inhibitors in periodontitis models. Additionally, exploring oligonucleotide-based targeted therapies (such as miRNA mimics, siRNA, ASOs) for predicted regulatory miRNAs (e.g., miR-1260b, miR-1224-5p) or lncRNAs (e.g., NEAT1) represents a crucial future direction.

This study is subject to several limitations. Chief among them is its reliance on retrospective analyses of publicly available transcriptomic datasets, which encompass limited sample sizes and restricted population diversity, thereby potentially constraining the generalizability of the findings. Future validation studies should incorporate well-powered, prospective multi-center clinical cohorts encompassing ethnically and geographically diverse populations and a spectrum of disease stages. Although bioinformatic inference and preliminary qRT-PCR validation (*n* = 5 per group) support the involvement of ABRHUB genes, the limited sample size and absence of functional assays constrain the strength of these conclusions. Mechanistic elucidation remains lacking—particularly regarding the precise roles of NEDD9 in TGF-β signaling and P2RX5 in calcium-mediated IL-1β induction. Comprehensive in vivo and in vitro functional assays—including gene knockout and overexpression models, co-culture systems, and pathway inhibition studies—are warranted to delineate these functional roles. Furthermore, the proposed regulatory interplay between miRNAs and lncRNAs necessitates stringent experimental corroboration using approaches such as dual-luciferase reporter assays, RNA immunoprecipitation, and RNA pull-down techniques. As a cross-sectional investigation, this study was unable to resolve the temporal dynamics of ABRHUB expression or its relationship with treatment responses, highlighting the imperative for longitudinally designed investigations. As a hypothesis-generating study, the ABRHUB identified herein should be viewed as candidate genes requiring rigorous experimental validation before any clinical translation can be considered.

## Conclusions

Through comprehensive bioinformatic interrogation, this study successfully identified NEDD9, NPR3, P2RX5 and CSF1R as candidate osteoclast-associated hub genes potentially implicated in periodontitis-induced alveolar bone resorption. These genes demonstrate promising diagnostic potential in silico and are associated with critical pathways—including TGF-β/SMAD, purinergic signaling, and CSF1R/NF-κB—as well as interactions with the immune microenvironment. However, as a hypothesis-generating study, these findings require rigorous experimental validation in future functional studies. Collectively, these genes represent promising candidates for further investigation as diagnostic biomarkers and therapeutic targets for periodontitis. Future research should prioritize: (1) validating their efficacy as non-invasive or minimally invasive diagnostic/prognostic biomarkers through qRT-PCR, immunohistochemistry, or the development of convenient detection kits (e.g., saliva/gingival crevicular fluid-based) in larger-scale, multi-center prospective cohorts; (2) constructing more accurate risk prediction models by integrating clinical indicators for the early identification of high-risk patients. The ultimate goal is to enable early, precise intervention and effective management of periodontitis, thereby alleviating the burden of this pervasive public health challenge.

## Materials and methods

### Data sources and processing

**Fig. 9:**
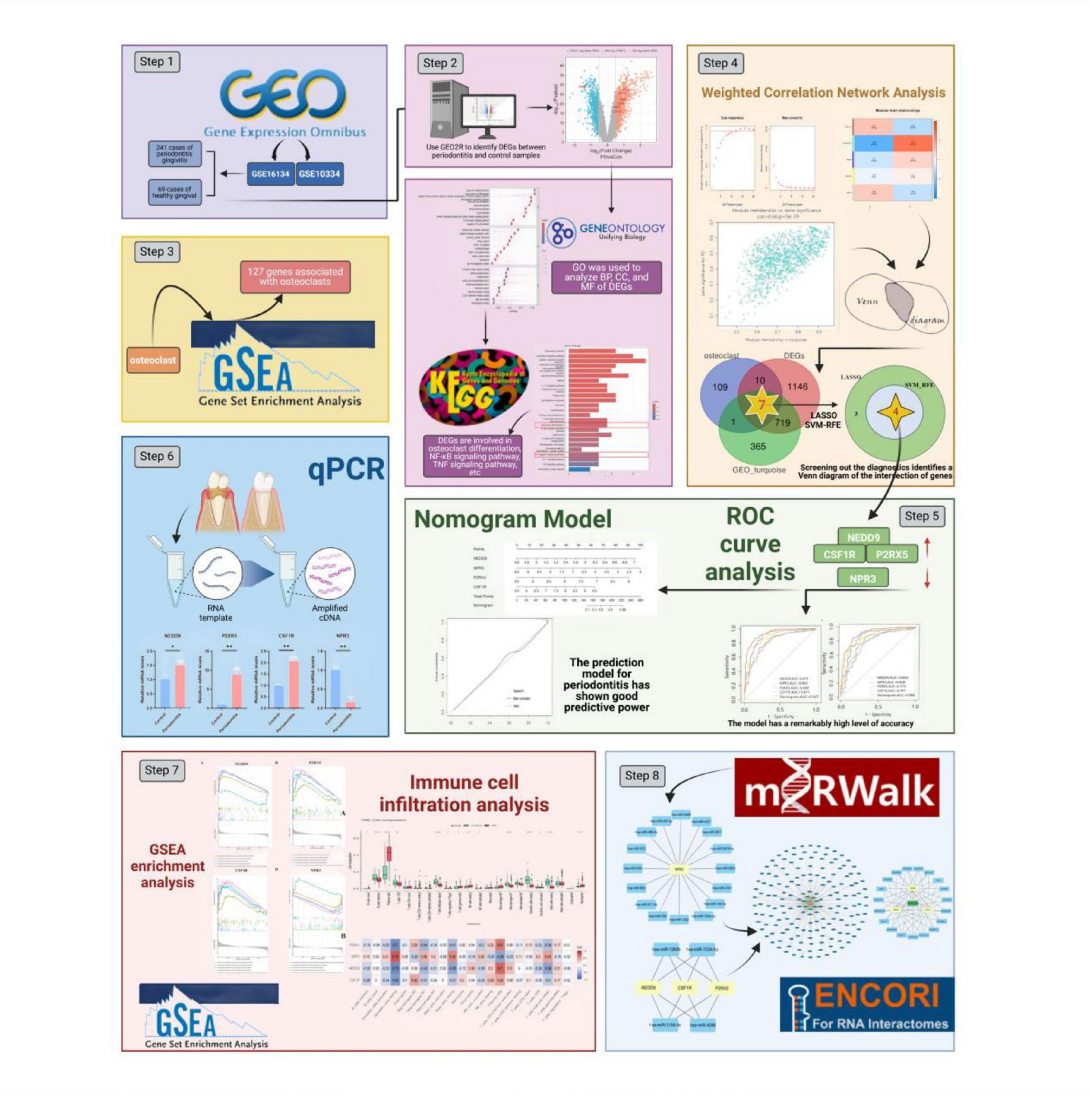
Flowchart of the analysis process.

The workflow of this study is illustrated in Fig. 9. The training and validation sets were obtained from the Gene Expression Omnibus (GEO; https://www.ncbi.nlm.nih.gov/geo/) maintained by the National Center for Biotechnology Information (NCBI). The inclusion criteria for dataset selection included: (1) comparative studies of gingival tissues from periodontitis patients versus healthy controls; (2) standardized high-throughput mRNA profiling via Affymetrix or Illumina platforms; (3) sufficient sample size; (4) raw data files. The GSE16134 dataset (GPL570 platform) was designated as the training cohort, comprising 241 PD samples and 69 healthy controls. The GSE10334 dataset (GPL96 platform) was used as an independent validation cohort, including 183 PD samples and 64 controls. To ensure data integrity, rigorous preprocessing of raw expression matrices was conducted in the R environment (v4.3.3), including: log2 transformation via the affy package (v1.80.0); probe-to-gene symbol conversion based on official annotation files, with median values used for multiple-probe mappings; and batch effect assessment and correction within the training cohort using the limma package (v3.58.1). Additionally, querying the Molecular Signatures Database (MSigDB; accessed March 20, 2024) with the term “osteoclast” retrieved 14 gene sets, of which 10 were pertinent to osteoclast development, proliferation, differentiation, and signaling cascades. Following the removal of duplicate entries, a curated set of 127 osteoclast-associated genes was compiled.

### Identification of differentially expressed genes

Differentially expressed genes (DEGs) between periodontitis and control samples were identified using GEO2R (http://www.ncbi.nlm.nih.gov/geo/geo2r). DEG was identified based on adjusted P-value < 0. 05 and |log fold change (FC)|>0. 5.

### GO annotation and KEGG pathway enrichment analysis

Gene Ontology (GO) annotation—including Biological Process (BP), Cellular Component (CC), and Molecular Function (MF)—as well as Kyoto Encyclopedia of Genes and Genomes (KEGG) pathway enrichment analyses were conducted on the DEGs identified in step 2 using the clusterProfiler package (v4.10.1) within the R environment (v4.3.3). Enrichment outcomes were considered statistically significant at a threshold of adjusted P-value < 0.05, following correction via the Benjamini–Hochberg procedure.

### Weighted gene co-expression network analysis

WGCNA was performed on the preprocessed gene expression matrix from the GSE16134 training cohort using the WGCNA package (v1.73) in R (v4.3.3). WGCNA was selected for its robust capacity to construct scale-free networks and identify co-expression modules and hub genes highly correlated with phenotypic traits, aligning with the study’s aim to systematically dissect the gene regulatory networks underlying periodontal bone resorption. Initially, low-quality genes and samples were filtered using the goodSamplesGenes function. Based on the analysis of network topology—specifically mean connectivity and scale-free topology fit—the soft-thresholding power (β) was determined to be 12 for constructing a scale-free adjacency matrix. This value was selected as it was the lowest power that achieved a scale-free topology fit index (R²) above 0.85, ensuring approximate scale-free network characteristics while maintaining sufficient mean connectivity. Module detection was conducted using dynamic tree cut with a minimum module size of 30. The correlation between module eigengenes and periodontitis was calculated to identify the most significantly associated module. Subsequently, Venn diagram analysis was performed using an online tool (http://bioinformatics.psb.ugent.be/webtools/Venn/) to intersect genes from WGCNA modules, osteoclast-associated gene sets from the Molecular Signature Database, and DEGs, thereby identifying the common differentially expressed genes (co-DEGs).

### Identification of ABRHUB

The Least Absolute Shrinkage and Selection Operator (LASSO) regression was applied to further refine the candidate genes associated with osteoclast activity and periodontitis. LASSO logistic regression analysis was performed using the glmnet package (v4.1–8) in R. LASSO was selected for its strength in handling high-dimensional data and its inherent ability to perform automatic feature selection, thereby minimizing overfitting and highlighting the most informative genes. The optimal regularization parameter (lambda) was determined through 5-fold cross-validation (nfolds = 5), using the lambda.1se criterion—defined as the largest lambda value within one standard error of the minimum cross-validation error—to select the most parsimonious model with predictive performance comparable to the best model. In addition, the support vector machine-recursive feature elimination (SVM-RFE) algorithm was implemented using the e1071 (v1.7–16) and caret (v6.0–94) packages in R. SVM-RFE was chosen for its efficacy in evaluating feature importance and recursively eliminating less informative variables, facilitating the identification of the optimal feature subset. The optimal feature subset size was determined by identifying the minimum cross-validation error during iterative model training. This recursive elimination approach sequentially removes the least important features, enabling identification of the minimal gene set that maximises classification accuracy. Genes commonly identified by both LASSO and SVM-RFE were selected as candidate ABRHUB genes.

### ABRHUB diagnostic value and risk modeling

Utilizing the GSE16134 training set and GSE10334 validation set, differential analysis was conducted for each ABRHUB to identify core genes that are significantly differentially expressed in periodontitis. To further enhance predictive power, the “rms” package (v6.8-1) was employed to construct a Nomogram risk model for periodontitis by integrating the differential genes and associated clinical features. This model integrates the results of multivariate regression analysis to identify independent risk factors. Ultimately, calibration curves were employed to evaluate the predictive power of the Nomogram model, thereby ensuring its reliability in clinical applications. Simultaneously, ROC curves were generated to evaluate the diagnostic efficacy of each ABRHUB in periodontitis, with the area under the curve (AUC) calculated to ascertain its diagnostic value. To reduce potential overfitting and enhance robustness, we performed bootstrap resampling (1,000 iterations) to compute 95% confidence intervals for the AUC values. ROC analysis was conducted using the R software package “pROC” (v1.18.5) to validate the model’s efficacy.

### Gene set enrichment analysis of ABRHUB

Gene set enrichment analysis (GSEA, v4.3.2) was used to identify potential molecular mechanisms of key genes. |NES|>1 and FDR < 0. 25 were considered as significant enrichment.

### Immune cell infiltration analysis

The infiltration levels of 22 immune cell subsets were quantified using the CIBERSORT algorithm (v1.03), which employs a support vector regression-based deconvolution method to estimate the relative fractions of immune cell types from bulk gene expression data. The analysis was performed with 1,000 permutations, and only samples achieving a CIBERSORT-derived deconvolution P-value < 0.05 were retained for subsequent analyses to ensure estimation reliability. The resulting immune cell fraction matrix was normalized to sum to 1 per sample. To visualize differences in immune infiltration between periodontitis and healthy control samples, heatmaps were generated using the “heatmap” package (v1.0.12) in R, and bar plots were created using “ggplot2” (v3.4.0). These visualizations enabled clear comparison of immune cell composition changes between groups. Furthermore, the relationship between ABRHUB expression and immune cell infiltration was assessed using Spearman’s rank correlation analysis, and the correlation matrix was visualized with the “corrplot” package (v0.94) in R to enhance interpretability of the results.

### Predicting targeted miRNAs/lncRNAs

The miRWalk database (http://mirwalk.umm.uni-heidelberg.de/) was used to predict the target miRNAs of ABRHUB. MiRNA dataset GSE54710 (adj *P* < 0. 05 and |logFC|>0. 5) from periodontitis patients obtained from GEO database was used for validation and the miRNAs that were predicted by the 2 databases simultaneously predicted miRNAs were considered to be target miRNAs of ABRHUB. MiRNAs targeting ABRHUB at the same time were considered to be key miRNAs affecting alveolar bone resorption in patients with periodontitis. Similarly, we further predicted miRNA-targeted lncRNAs using the ENCORI database (https://rnasysu.com/encori/). Based on the above predicted molecular regulatory relationships, Cytoscape software was used to visualize the prediction results and draw network diagrams.

### Clinical sample collection

The study was conducted in the Affiliated Hospital of North Sichuan Medical College and was approved by the Ethics Committee of the institution (No. 2024072), following the ethical standards set forth in the Declaration of Helsinki. The periodontal status of all participants was assessed by the same periodontist. The diagnosis of periodontitis followed the 2017 World Symposium definition of periodontitis cases. Patients in the control group were adjudged healthy by periodontal examination and x-ray. For the inclusion of periodontitis samples, the criteria included being between 18 and 60 years of age, being in good health and having stage II/III/IV periodontitis. Exclusion criteria, on the other hand, included smoking, presence of cardiovascular and respiratory diseases, diabetes mellitus, HIV infection, systemic inflammation, undergoing immunosuppressive chemotherapy and females during pregnancy or lactation. In addition, patients who had received periodontal treatment prior to the study or who had used antibiotics or anti-inflammatory drugs in the last three months were excluded, as well as patients who were unable to maintain good oral hygiene. A total of five periodontitis patients and five periodontally healthy individuals were included in the study. All control samples were obtained at the time of tooth extraction or crown lengthening surgery, while the case group was obtained at the time of tooth extraction or periodontal surgery. All gingival samples were obtained from the Affiliated Hospital of North Sichuan Medical College and were processed after obtaining informed consent from the patients. The excised gingival tissues were rinsed with saline, quickly transferred to tissue RNA preservation solution (RNALater) (Beyotime, R0118, Shanghai, China) and stored overnight at 4 °C and then stored at −80 °C for subsequent analysis.

### qRT-PCR was performed to verify the mRNA expression of ABRHUB in PD

Gingival tissues from 5 PD patients and 5 healthy donors were collected for qRT-PCR to confirm whether the expression of screened ABRHUB in PD patients was consistent with the results of the dataset. Extracted gingival tissues were preserved in RNA preservation solution. Standard procedures were followed with Trizol reagent (Ambion, Thermo Fisher Scientific, Waltham, MA, USA). The optical density ratio at 260 nm/280 nm was 1.8–2.0.8.0, which met the experimental requirements. RNA (1 µg) from each sample was reverse transcribed to cDNA using a 5×HiScript II Select qRT SuperMix II (VAZYME, Nanjing, China). The qPCR was performed with SYBR Green Master Mix (VAZYME) using a cfx96 Real-Time Fluorescent Quantitative PCR Detection System (Bio- Rad), programmed to perform a total of 40 cycles according to standard protocols. β-actin was used as an internal reference. Gene expression levels were estimated using the 2^−ΔΔCt^ method (see Table 1 for primer sequences). Statistical analysis was performed using t-test and *P* < 0. 05 was considered significant.(Table 1).

**Table 1:** ABRHUB primer sequence information.

gene name	Forward primer sequence (5’−3’)	Reverse primer sequence (5’−3’)
NEDD9	atggcaagggccttatatgaca	ttctgctctctatgacggtcagg
NPR3	agactacgccttcttcaacattg	gcttcaaagtcgtgtttgtctcc
P2RX5	ctgtcgctgttcgactacaag	cccatacgaccaggtacgc
CSF1R	gggaatccccagtgatagagcc	ttggaaggtagcgttgttgggt

### Statistical analysis

All data were calculated and statistically analyzed using the R language (v4.3.3). Between-group differences between independent variables were compared using t-tests. *p* < 0. 05 was considered statistically significant.

## Supplementary Information

Below is the link to the electronic supplementary material.

Supplementary Material 1

## Data Availability

The datasets generated and/or analyzed during the current study are available in the GEO repository (http://www.ncbi.nlm.nih.gov/geo/), GSE16134, GSE10334 and GSE54710.

## Abbreviations

ABRHUB: Alveolar bone resorption hub genes
WGCNA: Weighted gene co-expression network analysis
co-DEGs: Co-expressed differential genes
TNF-α: Tumor necrosis factor alpha
IL-1β: Interleukin-1β
ROS: Reactive oxygen species
ROC curve: Receiver operating characteristics curve
GEO: Gene Expression Omnibus
PD: Periodontitis
FC: Fold change
BP: Biological process
CC: Cellular component
MF: Molecular function
DEGs: Differentially expressed genes
LASSO regression: Least absolute shrinkage and selection operator regression
SVM-RFE algorithm: Support vector machine recursive feature elimination algorithm
AUC: Area under the curve
GSEA: Gene set enrichment analysis
GO: Gene ontology
KEGG pathway: Kyoto Encyclopedia of Genes and Genomes pathway
BMM: Bone marrow mononuclear cells
TGF-β: Transforming growth factor beta
CNP: C-type natriuretic peptide
ATP: Adenosine triphosphate
CSF1R: Colony stimulating factor 1 receptor
MAPK: Mitogen-activated protein kinase
PI3K: Phosphatidylinositol-3 kinase
Keap1: Kelch-like ECH-related protein 1
HO-1: Heme oxygenase-1
miRNAs: MicroRNAs
lncRNAs: Long-chain non-coding RNAs
